# Hyperlipidemia and Statin Use on the Progression of Osteoarthritis: A Systematic Review

**DOI:** 10.7759/cureus.15999

**Published:** 2021-06-28

**Authors:** Swetha Nukala, Suvarna Rekha Puvvada, Enkhmaa Luvsannyam, Dhara Patel, Pousette Hamid

**Affiliations:** 1 Department of Research, California Institute of Behavioral Neurosciences & Psychology, Fairfield, USA; 2 Department of Research, California Instititute of Behavioral Neurosciences & Psychology, Fairfield, USA; 3 Neurology, California Institute of Behavioral Neurosciences & Psychology, Fairfield, USA

**Keywords:** hyperlipidemia and osteoarthritis, lipids and osteoarthritis, hyperlipidemia and cartilage remodeling, metabolic syndrome and osteoarthritis, statins and osteoarthritis, hyperlipidemia and joints

## Abstract

Osteoarthritis (OA) is progressive wear and tear disease that affects multiple joints, including knees, hips, and spine. OA causes structural damage to joints. Although hypertension, type II diabetes, and hyperlipidemia have a silent effect, for the most part, the addition of osteoarthritis has a limiting and debilitating impact on patients. Common symptoms of OA include joint pain, joint swelling, limitations in range of motion that is impacting one’s quality of life. The question being investigated in this systematic review is whether hyperlipidemia or the use of statin has any effect on osteoarthritis and progression of osteoarthritis. This systematic review of 13 articles was done to explore if there is an effect of hyperlipidemia and statin use on the progression of osteoarthritis. This study included 6,974,538 total participants. Eight studies out of the 13 investigated the effect of statin use. Out of the 13 articles, five studies investigated the impact of lipid levels on OA. The total participants cannot be divided into statin users and non-users because some studies did not divide the participants into two groups. Studies that investigated the effect of lipid levels on OA were studied based on age. Some included only women in their study, and one study was solely conducted in the military population. Therefore, these studies cannot be divided based on age. Further research is needed to significantly conclude either the positive or negative correlation of hyperlipidemia and statin use on osteoarthritis and its progression.

## Introduction and background

A 92-year-old female patient with multiple chronic age-related conditions once gave me a prescription that stated, “Don’t grow up, stay young!” She said this prescription has lifelong refills and doesn’t expire. This caught my attention that, day in and day out, we encounter patients with a combination of common chronic conditions such as hypertension, type II diabetes mellitus, hyperlipidemia, and osteoarthritis (OA) that patients commonly attribute to age. Although hypertension, type II diabetes, and hyperlipidemia have a silent effect, for the most part, the addition of osteoarthritis has a limiting and debilitating impact on patients. It is reported that from 30 to 65 years of age, the prevalence and incidence of Osteoarthritis increases two to 10 times and keeps growing thereafter [1]. A meta-analysis reported a 30% prevalence of dyslipidemia with OA [2], partly owing to the underlying pro-inflammatory effect of dyslipidemia. It is said that there is a 1.46-fold increased risk of OA and diabetes mellitus [3]. Existing conditions such as hyperlipidemia, hypertension, and diabetes have an underlying pro-inflammatory effect on the joints. Research indicates a systemic influence among these chronic conditions, although there is no strong correlation among some of these.

Osteoarthritis, also known as degenerative joint disease, is progressive wear and tear disease that affects multiple joints, including knees, hips, and spine. OA causes structural damage to joints. It is reported that 80% of the population suffers from low back pain that is limiting and disabling [4], although not all causes of low back pain are due to OA. By age 70, the incidence of OA increases by 1% per year [1]. Common symptoms of OA include joint pain, joint swelling, limitations in range of motion that impacts one’s quality of life. As people age, the hyaline cartilage that cushions the joints recedes, eventually leading to narrowing of joint space that causes joints to abrade. This breakdown of the cartilaginous matrix is influenced by both anabolic components (e.g., Insulin-like growth factor I and II) and catabolic components (e.g., Tumor necrosis factor-alpha, Interleukin-1, and proteinases) [1]. The radiological changes include joint space narrowing, and bone spurs, although only 15% of patients with radiological changes actually complain of knee pain [1]. The diagnostic evaluation requires proper history, physical examination, and imaging studies. Early diagnosis leads to early intervention and prevention of the progression of the disease. Management includes pain control with conservative measures such as non-weight bearing exercise, non-steroidal anti-inflammatory drugs, opioid analgesics, and aggressive measures such as intraarticular injection, joint fluid therapy, epidural injections, and surgical intervention. Although OA is not yet curable, the best treatment is to prevent progression [1]. Risk factors that contribute to OA include age and obesity. Although there is no significant positive evidence, several research studies indicate that metabolic syndrome can contribute to the development and progression of OA [5]. One such metabolic risk factor is hyperlipidemia. Hyperlipidemia is elevated levels of lipids in the blood. It is an independent risk factor for cardiovascular diseases. Hyperlipidemia is primarily managed with diet, exercise, and the use of statins (hydroxyl-methyl-glutaryl coenzyme A reductase inhibitors) mainly. Management of hyperlipidemia is primarily cardioprotective.

Several research studies indicate that OA is not only associated with aging and mechanical stress but also with metabolic syndrome that contributes to the development and/ or progression of OA [5]. One such metabolic risk factor includes hyperlipidemia. In this article, we are exploring whether hyperlipidemia or the use of statin has any effect on osteoarthritis and the progression of osteoarthritis. We are writing a systematic review while conducting research from previous studies.

## Review

Methods

Search Strategy

The systematic review was validated using the Preferred Reporting Items for Systematic Reviews and Meta-Analyses (PRISMA) statement. The electronic database that was used to conduct this systematic review was PubMed and the MeSH strategy. Studies that were published between 2000 -2020 were included to explore whether hyperlipidemia or the use of statin has any effect on OA and its progression. The search strategy that was used were ("Osteoarthritis"[Mesh]) AND "Hyperlipidemias"[Mesh]; ("Hydroxymethylglutaryl-CoA Reductase Inhibitors"[Mesh]) AND "Osteoarthritis"[Mesh]; ("Hyperlipemia" OR "Hyperlipidemia" OR "Hyperlipidemias"[MeSH Terms] OR "Hyperlipidemias" OR "Lipemia" OR "Lipidemia") AND ("Arthritis, Degenerative" OR "Arthroses" OR "Arthrosis" OR "Osteoarthritis"[MeSH Terms] OR "Osteoarthritis" OR "Osteoarthrosis" OR "Osteoarthrosis Deformans") AND ("HMG-CoA Reductase Inhibitors" OR "Hydroxymethylglutaryl-CoA Reductase Inhibitors"[MeSH Terms] OR "Hydroxymethylglutaryl-CoA Reductase Inhibitors" OR "Inhibitors, HMG-CoA Reductase" OR "Inhibitors, Hydroxymethylglutaryl-CoA" OR "Inhibitors, Hydroxymethylglutaryl-Coenzyme A" OR "Statins" OR "Statins, HMG-CoA"); ("Dyslipidemias"[Mesh]) AND "Osteoarthritis"[Mesh]. Filters that were used include studies published in the past 20 years and written in the English language. 

*Study Selection* The inclusion includes 1) studies published between 2000-2020, 2) studies published globally, 3) studies published in the English language, 4) human studies, 5) randomized clinical trials, 6) non-randomized clinical trials, 7) observational studies, 8) systematic reviews.

The exclusion criteria include 1) letters, editorials, expert opinions, 2) animal studies, 3) In-vivo and In-vitro studies, 4) articles written in different languages 5) studies published more than 20 years ago.

*Screening and Data Collection* Each full-text article and abstract was assessed carefully for eligibility and relevance to the topic. The studies and abstracts that were not relevant were excluded. Animal studies, letters, expert opinions, and in-vivo and in-vitro studies were all excluded. The population of the studies includes males and females from various parts of the world, such as the US, UK, and Taiwan. The data collection included studies conducted globally.

*Quality Assessment* The quality of the studies included was evaluated. All randomized clinical trials were assessed using the Cochran risk bias assessment tool. All the observational studies and the non-randomized clinical trials were assessed using the New Cassel Ottawa scale. The systematic reviews were evaluated using the AMSTAR (Assessment of multiple systematic reviews) checklist. The reporting guidelines used were the PRISMA guidelines. 

Results

**Figure 1:**
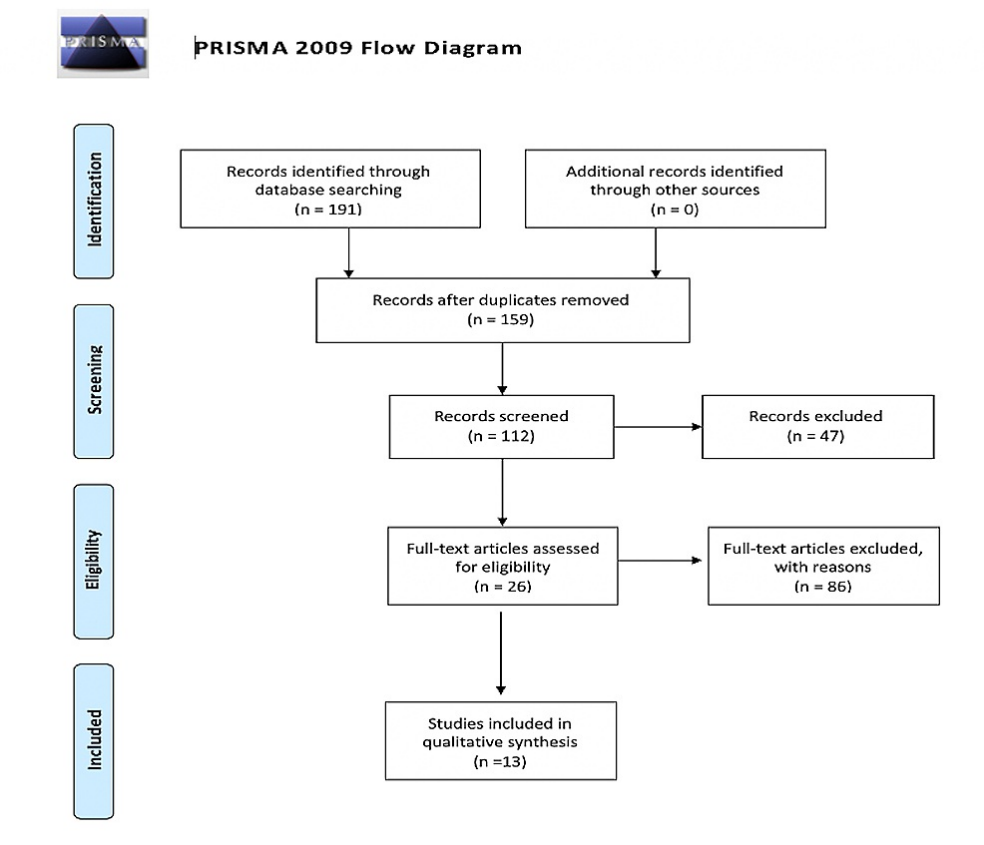
PRISMA Flow Diagram PRISMA (Preferred Reporting Items for Systematic Reviews and Meta-Analyses)

*Search Results* The initial search criteria included six regular keywords and three Mesh keywords that yielded 191 articles, as shown in Figure 1. After removing duplicates, 159 articles were screened using the title and abstract, and 47 were excluded. Out of 112 articles, 26 full articles were reviewed for relevance. Out of 26 full-text articles, 13 articles were excluded because they did not meet the inclusion criteria, resulting in 13 articles that were evaluated for quality assessment. This systematic review included 11 cohort studies, one case-control study, one systematic review, and a meta-analysis. Study characteristics of these 13 articles are recorded in Table 1.

*Study Characteristics* The study characteristics of each study were recorded in Table 1. It includes the author's name, publishing year, the type of study design, age of the participants that were studied, the study variables that were studied, sample size, and the years of follow-up. 

**Table 1:** Table of Characteristics DJD, Degenerative Joint Disease; OA, Osteoarthritis; MSK, Musculoskeletal.

Author, year	Study design	Location	Age of Participants	Study variables	Sample Size	Mean follow up in years
Cheng, et al. 2017 [6]	Cohort Study	Taiwan	Men and women between 40-65 years old between 2001-2010	Statin vs spinal DJD	7,238 statin users and 164, 454 non-users.	7 years
Zhou, et a., 2017 [7]	Cohort Study	China	Men and women with an average age of 64.7	Lipid levels vs Knee OA	13,906 middle-aged or older	N/A
Garcia-Gil, et al. 2017 [8]	Cohort Stud	UK	Women between 45-64 years	Lipid levels vs Hand OA	10,003 women	10 years
Frey, et al. 2017 [9]	Case-control Study	UK	Men and women `between 30-89 years	Lipid levels vs Hand OA	19,590 cases and 19,590 controls	N/A
Haj-Mirzaian, et al. 2019 [10]	Cohort Study	N/A	Women with an average age of 64.7	Statin vs Knee OA	602 total participants	8 years
Veronese, et al. 2019 [11]	Cohort Study	USA	Men and women with a mean age of 61.1	Statin vs Knee OA	4,448 total participants	4 years
Burkard, et al. 2018 [12]	Cohort Study	UK	Men and women between 45-84	Statin vs Hand OA	237,864 statin users and 6,020,144 non-users	5.5 years
Makris, et al. 2018 [13]	Cohort Study	USA	Men and women with a mean age of 52	Statin vs MSK	6,728 statin users with 6,728 nonusers	4 years
Michaelsson, et al. 2017 [14]	Cohort Study	Sweden	Men and women between 57-91 years of age of central Sweden	Statin vs hip or knee OA	132,607 total participants	7.5 years
Kadam, et al. 2013 [15]	Cohort Study	UK	Men and women over the age of 40	Statin vs clinic OA	16,609 total participants	2, 4 and 10 years
Clockaerts, et al. 2011 [16]	Cohort Study	Netherlands	Men and women over the age of 55	Statin vs hip and knee OA	7,983 total participants	6.5 years
Baudart, et al. 2017 [2]	Systematic Review and Meta-analysis	Global	Men and women over the age of 18	Lipid levels vs OA	306,044 total participants	N/A
Yoshimura, et al. 2012 [17]	Cohort Study	Japan	No age range reported	Lipid levels vs knee OA	1,384 total participants	3 years

*Studies* Each study was thoroughly evaluated. The outcome measures of each of them are recorded in Table 2 and Table 3. Table 2 portrays studies that investigated the effect of lipid levels on OA. Table 3 depicts studies that examined the effect of statin use on OA.

**Table 2:** Lipid levels vs type of OA KOA, Knee Osteoarthritis; RHOA, Radiographic Hand Osteoarthritis; LDL, Low-Density Lipid; HDL, High-Density Lipid; HOA, Hand Osteoarthritis.

Author	Age of participants	Inclusion criteria	Type of OA	Outcome
Zhou, et al. [7]	Participants with an average age of 64.7 years	13,906 middle-aged or older participants from the Dongfeng-Tongji cohort	Knee	Hyperlipidemia is associated with elevated risks of knee pain and clinical KOA among middle-aged or older adults.
Garcia-Gil, et al. [8]	Women between 45-64 years	All women in a prospective population-based cohort from the Chingford study with available baseline lipid measurements and without RHOA on a baseline.	Hand	No relationship was found with total or LDL cholesterol. Higher levels of HDL cholesterol appear to protect against RHOA after 11 years of follow-up
Frey, et al. [9]	Men and women between 30 and 89 years	Patients aged 30-89 years with a first-time recorded READ-code for HOA (according to ICD-10 M19.04) between January 1995 and December 2014, and with 3 years of HOA-free history prior to the first recorded HOA diagnosis	Hand	The association between HOA and hyperlipidemia is inversely correlated with increasing age
Baudart, et al. [2]	Men and women over the age of 18	306044 participants were included in the study.	Generalized	The risk of dyslipidemia was twofold greater in patients with OA than without OA. Lipid disturbances could be a risk factor for OA.
Yoshimura, et al. [17]	No age range reported	1384 participants were included in the study	Knee	The prevention of metabolic syndrome may be useful in reducing future KOA risk.

**Table 3:** Statin use vs type of OA HN, Heberden Nodes; UK, United Kingdom; OA, Osteoarthritis

Author	Participants	Inclusion criteria	Type of OA	Outcome
Cheng, et al. [6]	Participants between 40-65 years old	7238 statin users and 164, 454 non-users were identified and followed up for the next 7 years.	Spinal DJD	In patients with hypercholesterolemia, a higher dosage of statins can reduce the incidence of spinal degenerative joint disease.
Haj-Mirzaian, et al. [10]	Women with an average age of 64.7	Osteoarthritis Initiative cohort was used to conduct a longitudinal 1:1 propensity score-matched retrospective analysis of prospectively collected data. Participants were classified as having HN-positive or HN-negative findings according to the presence of HNs	Knee	In patients with nodal OA, statin use was linked to a reduced risk of progression of radiographic knee osteoarthritis joint space narrowing.
Veronese, et al. [11]	Men and women with a mean age of 61.1	A total of 4,448 adults from the Osteoarthritis Initiative were followed up for 4 years.	Knee	In individuals using statins for >5 years and those using atorvastatin specifically had a significantly lower risk of developing knee pain
Burkard, et al. [12]	Men and women between 45-84	Patients between 45-84 years of age who were in the UK-based Clinical Practice-Based Research Datalink were extracted. Those patients with >1 new prescription for atorvastatin, fluvastatin, pravastatin, rosuvastatin, or simvastatin after a statin free period of at least 3 years were included in the study	Hand	There was no association between initiation of statin and incidence of hand OA.
Makris, et al. [13]	Men and women with a mean age of 52	Men and women enrolled in the military healthcare system were evaluated. score-matched 6728 statin users and 6728 nonusers were included in the study	Generalized	Statin use was associated with a significantly increased risk of non-traumatic arthropathies and use-related injury.
Michaelsson, et al. [14]	Men and women between 57-91 years of age of central Sweden	The association between statin use and time to consultation or surgery for OA of the hip or knee was studied in the 4 different population cohorts.	Hip and Knee	Statin use is not associated with a reduced risk of consultation or surgery for OA of the hip or knee.
Kadam, et al. [15]	Men and women over the age of 40	16,609 adults with cardiovascular disease cohorts from the UK General Practice Research Database with data available to 31 December 2006 were included in the study	Generalized	There was a significant reduction in clinical OA outcome with the use of higher statin dose and larger statin dose increments with a treatment duration of >2 years.
Clockaerts, et al. [16]	Men and women over the age of 55	7983 participants were included in the study. Participants with radiographs of knee and hip were included and compared over the years.	Hip and knee	With statin use, there was more than a 50% reduction in the overall progression of osteoarthritis of the knee, but not of the hip.

Discussion

Osteoarthritis is a progressive joint condition that is possibly affected by uncontrolled lipid levels. Further research is necessary to make a significant correlation. Treatment of OA is currently dependent on preventing progression to better the lifestyle of affected patients. Treatment includes symptomatic relief such as analgesics, physical therapy, corticosteroid injections, and surgical intervention as a last resort.

This systematic review includes 13 articles that studied 6,974,538 total participants. The two aspects that were investigated include the effect of hyperlipidemia and statin use on OA and its progression. Eight studies out of the 13 investigated the effect of statin use. Out of the 13 articles, five studies investigated the effects of lipid levels on OA. The total participants cannot be divided into statin users and non-users because some studies did not break the participants into two groups. Studies that investigated the effect of lipid levels on OA were studied based on age. Some included only women in their research, and one was solely in the military population. Therefore, these studies cannot be divided based on age. 

Lipid Levels vs. OA

*Lipid Levels vs. Knee OA* Zhou, et al. and Yoshimura, et al. both analyzed the effects of lipid levels on Knee OA. Zhou, et al. reported that with every one unit increase in triglycerides, there were an associated 9% and 5% increases in the risk of clinical KOA prevalence and clinical KOA onset, respectively [7]. Therefore, among middle-aged and older adults, hyperlipidemia is associated with elevated risks of knee pain and clinical presentation of KOA. Similarly, Yoshimura, et al. reported that the accumulation of metabolic syndrome components is significantly related to both occurrence and progression of KOA. Prevention of metabolic syndrome may be useful in reducing the risk of future KOA [17]. Although both studies reported similar results, the differences include the sample size and study population. The sample size studied by Zhou, et al. was significantly larger than the sample size of Yoshimura, et al.

*Lipid Levels vs. Hand OA* Garcia-Gill, et al. and Frey, et al. both investigated the effect of lipid levels on hand OA. Garcia-Gill, et al. included all women in a prospective population-based cohort from the Chingford study with available baseline lipid measurements and without RHOA (Radiographic Hand Osteoarthritis) on a baseline. This resulted in no relationship between total or LDL (Low-Density Lipid) cholesterol and hand OA, but after 11 years of follow-up, higher levels of HDL (High-Density Lipid) cholesterol appear to be protective against RHOA [8]. The study conducted by Frey, et al. included patients with a first-time recorded READ-code for HOA (Hand Osteoarthritis; according to ICD-10 M19.04) and with three years of HOA-free history prior to the first recorded HOA diagnosis. They reported that the association between HOA and hyperlipidemia is inversely correlated with increasing age [9]. Although both studies yielded in similar results, there is a difference in the study aspect regarding population age and gender. The study design of Frey, et al. is broad in terms of the age of the population as well as the study group which consisted of both men and women. In contrast, the study population included by Garcia-Gill, et al. is only women between 45 and 64 years of age.

*Lipid Levels vs. Generalized OA* Baudart, et al. conducted a systematic review of 48 articles that included a total of 306044 participants. They reported that the risk of dyslipidemia was twofold greater in patients with OA than in patients without OA, leading to lipid disturbances being a risk factor for OA [2].

Statin use vs. OA

*Statin Use vs. Knee OA* Haj-Mirzaian, et al. and Veronese, et al. analyzed the effect of statin use on knee OA. The study population of Haj-Mirzaian, et al. included women with a mean age of 67.7. They used the Osteoarthritis Initiative cohort to conduct a longitudinal 1:1 propensity score-matched retrospective analysis of prospectively collected data. Participants were classified as having Heberden Node-positive or Heberden Node-negative. The study reported that statin use was linked to a reduced risk of progression of radiographic knee osteoarthritis joint space narrowing in patients with nodal OA [10].

On the other hand, Veronese, et al. included men and women with an average age of 61.1. A total of 4,448 adults from the Osteoarthritis Initiative study were followed up for four years. In individuals using statins for more than five years and those using atorvastatin specifically, there was a significantly lower risk of developing knee pain [11].

*Stain Use vs. Hip and Knee OA* Michaelsen, et al. and Clockaerts, et al. studied the effect of statin use on hip and knee OA. Michaelsen, et al. included men and women between 57-91 years of age in central Sweden. They researched the association between statin use and the time to consultation to surgery for OA of the hip or knee in four different population cohorts. They concluded that statin use is not associated with reduced risk of consultation or surgery for OA of the hip or knee [14]. On the other hand, Clockaerts, et al. studied men and women over the age of 55. They included 7983 total participants who were compared over the years. They reported that with statin use, there was a more than 50% reduction in the overall progression of osteoarthritis of the knee but not of the hip [16]. *Stain Use vs. Generalized OA* Makris, et al. and Kadam, et al. both investigated the correlation of statin use on generalized OA. Makris, et al. included men and women with a mean age of 52 who were enrolled in the military healthcare system. They included score-matched 6728 statin users with 6728 nonusers, leading to a conclusion that statin use was associated with a significantly increased risk of non-traumatic arthropathies and use-related injury [13]. On contrary, the study conducted by Kadam, et al. is different in terms of the study population. They included men and women over the age of 40. The study included 16,609 adults with cardiovascular disease cohorts enrolled in the UK General Practice Research Database with data available to December 31, 2006. There was a significant reduction in clinical OA outcomes with the use of higher statin dose and larger statin dose increments with a treatment duration of more than two years [15].

*Statin Use vs. Hand OA* Burkard, et al. analyzed the effect of statin use on hand OA. They included men and women between 45-84 years of age from the UK-based Clinical Practice-Based Research Datalink. Patients with more than one new prescription for atorvastatin, fluvastatin, pravastatin, rosuvastatin, or simvastatin after a statin-free period of at least three years were included. They concluded that there was no association between initiation of statin and incidence of hand OA [12].

*Statin Use vs. Spinal DJD (Degenerative Joint Disease)* Cheng, et al. explored the effect of statin use on spinal DJD. The study population included patients between the age of 40 and 65 years of age. They included 7238 statin users and 164 454 non-users and they were followed up for the next seven years. They concluded that a higher dosage of statins can reduce the incidence of spinal degenerative joint disease in patients with hypercholesterolemia [6]. 

Further investigation

There is adequate research supporting the effects of metabolic syndrome on the cardiovascular system. There is research that shows how weight gain affects the joints and how weight gain is related to hyperlipidemia. There is also research showing the effect of statins on the muscles. However, research is scarce on how metabolic syndrome affects OA and its progression. The questions that need further investigation include the effect of hyperlipidemia and statin use in women vs. men, young vs. elderly. Research is scarce on how it affects each age group and gender group. Not all studies that were included in this systematic review differentiated their sample sizes into males and females or by age. 

Limitations

This systematic review has some limitations. Even though global studies were included, the investigation was only conducted in English. Therefore, research studies conducted in different languages were excluded. Studies conducted before the year 2000 were also excluded. Overall, the articles that were published are finite, and additional data is required to make a significant difference.

## Conclusions

Osteoarthritis is progressive wear and tear disease that affects multiple joints by causing structural damage to joints. Although there is no validated positive conclusion, research indicates that metabolic syndrome can affect the development and progression of OA. One such metabolic risk factor is hyperlipidemia. This systematic review of 13 articles was done to explore if there is an effect of hyperlipidemia and statin use on the progression of osteoarthritis. Although hyperlipidemia can be a risk factor, it cannot be significantly concluded whether hyperlipidemia and the use of statin are impacting osteoarthritis since further research is needed to significantly conclude either the positive or negative correlation of hyperlipidemia and statin use on osteoarthritis and its progression. The research is limited due to research being scarce, limiting the search to only including articles that were published in English and excluding articles published before the year 2000.
